# Socio-demographic, health-related and psychosocial correlates of fear of falling and avoidance of activity in community-living older persons who avoid activity due to fear of falling

**DOI:** 10.1186/1471-2458-9-170

**Published:** 2009-06-02

**Authors:** Gertrudis IJM Kempen, Jolanda CM van Haastregt, Kevin J McKee, Kim Delbaere, GA Rixt Zijlstra

**Affiliations:** 1 School for Public Health and Primary Care, Faculty of Health, Medicine and Life Sciences, Maastricht University, The Netherlands; 2 Sheffield Institute for Studies on Ageing, University of Sheffield, Sheffield, UK; 3 Falls and Balance Research Group, Prince of Wales Medical Research Institute, University of New South Wales, Randwick, Sydney, Australia; 4 Department of Experimental-Clinical and Health Psychology, Faculty of Psychology and Educational Sciences, Ghent University, Ghent, Belgium; 5 Department of Rehabilitation Sciences and Physiotherapy, Faculty of Medicine and Health Sciences, Ghent University, Ghent, Belgium

## Abstract

**Background:**

Fear of falling and avoidance of activity are common in old age and are suggested to be (public) health problems of equal importance to falls. Earlier studies of correlates of fear of falling and avoidance of activity did hardly differentiate between severe and mild levels of fear of falling and avoidance of activity which may be relevant from clinical point of view. Furthermore, most studies focused only on socio-demographics and/or health-related variables and hardly incorporated an extensive range of potential correlates of fear of falling including psychosocial variables. This study analyzes the univariate and multivariate associations between five socio-demographic, seven health-related and six psychosocial variables and levels of fear of falling and avoidance of activity in older persons who avoid activity due to fear of falling.

**Methods:**

Cross-sectional study in 540 community-living older people aged ≥ 70 years with at least mild fear of falling and avoidance of activity. Chi-squares, t-tests and logistics regression analyses were performed to study the associations between the selected correlates and both outcomes.

**Results:**

Old age, female sex, limitations in activity of daily living, impaired vision, poor perceived health, chronic morbidity, falls, low general self-efficacy, low mastery, loneliness, feelings of anxiety and symptoms of depression were identified as univariate correlates of severe fear of falling and avoidance of activity. Female sex, limitations in activity of daily living and one or more falls in the previous six months correlated independently with severe fear of falling. Higher age and limitations in activity of daily living correlated independently with severe avoidance of activity.

**Conclusion:**

Psychosocial variables did not contribute independently to the difference between mild and severe fear of falling and to the difference between mild and severe avoidance of activity due to fear of falling. Although knowledge about the unique associations of specific variables with levels of severe fear of falling and avoidance of activity is of interest for theoretical reasons, knowledge of univariate association may also help to specify the concepts for developing interventions and programmes to reduce fear of falling and avoidance of activity in old age, particularly in their early stages of development.

## Background

Fear of falling and avoidance of activity due to fear of falling are common in older people. Prevalence rates for fear of falling in community-living older persons range from 20 to above 60% [1-8] and for avoidance of activity due to fear of falling from 15 to above 55% [1,6,7,9-11]. Fear of falling, and related avoidance of activity may lead to adverse consequences, like functional decline [8,12,13], restriction of social participation [9], decreased quality of life [2,8,12], increased risk of falling [8,10] and institutionalization [12], and will have societal implications related to health and social care utilization and associated costs. Indeed, fear of falling is suggested to be a potential (public) health problem of equal importance to a fall [10].

Previous research has shown that fear of falling and/or avoidance of activity were associated with sociodemographic factors (i.e., age, sex and living alone status), health-related variables (perceived health problems, cognitive impairment, physical function and falls history) and psychosocial variables (i.e., symptoms of anxiety and depression) [2,3,6-8,13-18]. Amongst older people with fear of falling, females, those with greater frailty, more chronic conditions and limitations, lack of support, and higher levels of depression are more likely to restrict activity [5,6].

Zijlstra and colleagues [7], in reviewing the research literature found that factors independently related to fear of falling and particularly to avoidance of activity due to fear of falling are clearly under researched. While there have been previous studies (see above), most studies focused only on socio-demographics and/or health-related variables and hardly any study incorporated an extensive range of potential correlates of fear of falling including psychosocial variables, which would enable the independent contribution of specific variables to be determined. In addition, hardly any study differentiated between *severe *and *mild *levels of fear of falling and avoidance of activity which might be relevant from *clinical *point of view. Knowledge of these factors may facilitate identification of those older persons who are suffering from severe levels of fear of falling and avoidance of activity, and thereby offer routes to effective intervention.

The present cross-sectional study was performed in a sample of community-living older people in the Netherlands who reported at least some fear of falling and associated avoidance of activity. The objective was to examine the relationship between severe levels of fear of falling and avoidance of activity on the one hand, and an extensive range of potential correlates on the other, including socio-demographic, health-related and psychosocial variables.

## Methods

### Design

The study was of cross-sectional design and used the baseline data of a trial evaluating the effects of a cognitive behavioral group intervention to reduce fear of falling and fear-related activity avoidance among community-living older persons [19].

### Participants

Between November 2002 and July 2003, local municipal registry offices of two cities in the south of the Netherlands selected a random sample of 7,431 community-living people aged 70 years and over. They received a short screening questionnaire [19], which 4,376 people (59%) returned. Respondents matching the following inclusion criteria were included in the trial: 1) reporting at least mild fear of falling; 2) reporting at least mild avoidance of activity due to fear of falling; 3) living in the community; and 4) being at least 70 years old [19]. We included 540 community-living people aged 70 years and over who reported at least mild fear of falling and at least mild avoidance of activity due to this fear. We excluded people who were bedridden, wheelchair-bound, or on the waiting list for admission to a nursing home. A more detailed description of the recruitment procedure is available elsewhere [14,19].

### Measures

Fear of falling was assessed by asking "Are you afraid of falling?", and avoidance of activity due to fear of falling was measured by asking "Do you avoid certain activities due to fear of falling?" Possible answers to these questions were: "never", "almost never", "sometimes", "often", or "very often". For the mentioned trial we included only participants who reported either "sometimes", "often" or "very often" on both questions. The answer option "sometimes" was denoted as mild fear of falling and avoidance of activity due to this fear. The answer options "often" and "very often" was denoted as severe fear of falling and avoidance of activity due to this fear.

Three groups of correlates were assessed: 1) socio-demographic, 2) health-related and 3) psychosocial correlates.

First, we assessed the following socio-demographic variables: age, sex, educational level and living arrangements. Educational level was based on all schooling and professional courses completed in the course of life (lower versus middle/high) and living arrangement was assessed as living alone or living with one or more others.

Second, the health-related variables we assessed were limitations in activities of daily living, impaired vision and hearing, perceived general health, number of chronic conditions, cognitive functioning and falls history. Limitations in activities of daily living were assessed with the 11-item subscale of the Groningen Activity Restriction Scale (GARS; theoretical score range 11–44) [20]. Impaired vision and hearing were each measured with two items according to the indicator of the Organization for Economic Co-operation and Development (score range for both measures 2–8) [21]. Perceived general health was measured with item one of the MOS SF-20 (poor/fair versus good/very good/excellent) [22]. Number of medical conditions was assessed with a checklist of 19 chronic medical conditions which was used by the Dutch national office for statistics (score range 0–19) [23]. Cognitive functioning was assessed with the 25-item Dutch version of the Telephone Interview for Cognitive Status (TICS; score range 0–41) [24]. Falls history was assessed with a single item: how frequently have you fallen in the past six months [19].

Lastly, the psychosocial variables assessed were general self-efficacy, mastery, social support interactions, feelings of loneliness, and feelings of anxiety and symptoms of depression. General self-efficacy was assessed with a 16-item general self-efficacy questionnaire (score range 16–80) [25]. Mastery was assessed with a 7-item mastery scale (score range 7–35) [26]. Frequency of social support interactions was assessed with the 12-item Social Support Scale for Interactions (score range 12–48) [27]. Feelings of loneliness were assessed by the question 'During the past four weeks, how often did you feel lonely?' (1 = all the time to 6 = never; dichotomized in: all the time/mostly/often versus sometimes/almost never/never). Feelings of anxiety and symptoms of depression were measured with the 14-item Hospital Anxiety and Depression Scale (HADS) [28]. The HADS can be divided into subscales for "feelings of anxiety" (7 items; score range 7–28) and "symptoms of depression" (7 items; score range 7–28).

Chronic medical conditions, TICS, GARS and impaired vision and hearing were assessed by telephone interviews. All other data were collected by means of self-administered questionnaires. The study was approved by the medical ethics committee of Maastricht University/Academic Hospital in Maastricht, the Netherlands.

### Statistical analysis

First, descriptive analyses were performed for all selected variables. Second, we performed chi squares and independent t-tests to study univariate associations between the socio-demographic, health-related and psychosocial variables on the one hand and the two levels of fear of falling (mild and severe) on the other. We then repeated the same analyses for avoidance of activity. Finally, we conducted logistic regression analyses for each of the two dependent variables: fear of falling and avoidance of activity. Only the socio-demographic, health-related or psychosocial variables that showed a univariate association of p < .05 were included simultaneously in these analyses. We estimated odds ratios (ORs) and corresponding 95% confidence intervals (CIs). Analyses were conducted using SPSS for Windows version 14.0 (SPSS, Inc. Chicago, IL). A significance level of less than .05 was considered statistically significant.

## Results

Table 1 presents the characteristics of the 540 participants in the study. The majority of the participants were female, with low level of education, and lived alone. Nearly 70% of participants perceived their health as fair to poor. Furthermore, 55% reported one or more falls in the previous 6 months. Finally, 45% of participants reported severe fear of falling and 42% severe avoidance of activity due to this fear.

**Table 1:** Characteristics of the participants (n = 540)

*Socio-demographic*			
-Age (mean, SD, range)	77.6	4.8	70 – 92
-Female (n, %)	388	72%	
-Low education (n, %)^1^	338	63%	
-Living alone (n, %)	295	55%	
			
*Health-related*			
-Activity of daily living (mean, SD, range)^2^	17.3	4.5	11 – 40
-Impaired vision (mean, SD, range)^2^	2.9	1.5	2 – 8
-Impaired hearing (mean, SD, range)^2^	2.8	1.1	2 – 7
-Poor/fair perceived general health (n, %)	375	69%	
-Number of chronic conditions (mean, SD, range)	2.3	1.6	0 – 9
-Cognitive functioning (mean, SD, range)^3^	32.0	3.8	16 – 40
-One or more falls in previous six months (n, %)	298	55%	
			
*Psychosocial*			
-General self-efficacy (mean, SD, range)^3^	54.2	11.1	16 – 80
-Mastery (mean, SD, range)^3^	21.2	4.6	9 – 35
-Social support interactions (mean, SD, range)^3^	29.5	6.7	12 – 48
-Feelings of loneliness (n, % all the time/mostly/often)	91	17%	
-Feelings of anxiety (mean, SD, range)^2^	7.4	4.5	0 – 21
-Symptoms of depression (mean, SD, range)^2^	6.8	4.0	0 – 19
			
*Fear of falling*			
- Mild (n, %)	298	55%	
- Severe (n, %)	242	45%	
			
*Avoidance of activity*			
- Mild (n, %)	315	58%	
- Severe (n, %)	225	42%	

^1 ^Low education refers to elementary school, junior high school or lower vocational level. ^2 ^Higher level refers to poorer functioning. ^3 ^Higher level refers to better functioning

Table 2 summarizes the outcomes of the univariate analyses of the socio-demographic, health-related and psychosocial variables on the one hand and fear of falling and avoidance of activity on the other. All selected variables were significantly associated with fear of falling in the expected direction except for impaired hearing, cognitive dysfunctioning and social support interactions. There was also no significant association between these latter variables and avoidance of activity due to fear of falling, which was in addition not significantly associated with low education and living alone status.

**Table 2:** Univariate associations between socio-demographic, health-related and psychosocial variables and fear of falling and avoidance of activity

	Fear of falling		Avoidance of activity	
	Mild (n = 298)	Severe (n = 242)	Mild (n = 315)	Severe (n = 225)
		
*Socio-demographic*				
-Age (M, SD)	77.2 (4.6)	78.2 (4.9)*	77.1 (4.7)	78.3 (4.8)*
-Sex (female)	64.8%	80.6%**	67.9%	77.3%**
-Educational level (low)^1^	59.4%	66.8%**	59.9%	66.7%
-Living alone	50.0%	60.3%**	52.4%	57.8%
				
*Health-related*				
-Activity of daily living (M, SD)^2^	15.9 (3.5)	19.0 (4.9)*	15.8 (3.4)	19.4 (5.0)*
-Impaired vision (M, SD)^2^	2.7 (1.3)	3.1 (1.6)*	2.7 (1.3)	3.1 (1.7)*
-Impaired hearing (M, SD)^2^	2.7 (1.1)	2.8 (1.1)	2.7 (1.0)	2.8 (1.1)
-Perceived general health (poor/fair)	64.9%	75.3%**	63.3%	78.4%**
-Number of chronic conditions (M, SD)	2.2 (1.5)	2.5 (1.7)*	2.1 (1.5)	2.6 (1.7)*
-Cognitive functioning (M, SD)^3^	32.1 (3.8)	31.8 (3.7)	32.1 (4.0)	31.7 (3.4)
-One or more falls in previous six months	49.0%	63.6%**	50.3%	62.9%**
				
*Psychosocial*				
-General self-efficacy (M, SD)^3^	55.8 (10.2)	52.3 (11.9)*	55.6 (10.4)	52.4 (11.9)*
-Mastery (M, SD)^3^	21.7 (4.5)	20.6 (4.7)*	21.9 (4.7)	20.2 (4.4)*
-Social support interactions (M, SD)^3^	29.5 (6.3)	29.5 (7.3)	29.9 (6.3)	29.1 (7.3)
-Feelings of loneliness (all the time/mostly/often)	13.5%	21.3%**	12.5%	23.4%**
-Feelings of anxiety (M, SD)^2^	6.5 (4.1)	8.4 (4.8)*	6.6 (4.2)	8.5 (4.7)*
-Symptoms of depression (M, SD)^2^	6.1 (3.6)	7.7 (4.2)*	6.3 (3.7)	7.5 (4.2)*

p < 0.05 * T-test ** Chi-square

^1 ^Low education refers to elementary school, junior high school or lower vocational level. ^2 ^Higher level refers to poorer functioning. ^3 ^Higher level refers to better functioning

The outcomes of the logistic regression analyses are presented in Table 3. Only the significant univariate correlates (see Table 2) were included. When all significant univariate correlates are entered in the regression equation simultaneously, female sex, limitations in activity of daily living and one or more falls in the previous six months correlate independently with severe fear of falling. With respect to severe avoidance of activity due to fear of falling, higher age and limitations in activity of daily living correlate independently.

**Table 3:** Logistic regression analysis: odds ratios and 95% confidence intervals for fear of falling and avoidance of activity for socio-demographic, health-related and psychosocial variables

	Fear of falling		Avoidance of activity	
	Mild	Severe	Mild	Severe
		
	OR 95% CI	OR 95% CI	OR 95% CI	OR 95% CI
*Socio-demographic*				
-Age	Reference	1.04 (1.00 – 1.08)	Reference	1.04 (1.00 – 1.09)
-Sex (female)	Reference	2.28 (1.41 – 3.69)	Reference	1.40 (0.89 – 2.19)
-Educational level (low)^1^	Reference	1.03 (0.68 – 1.56)	--- -------------	--- -----------
-Living alone	Reference	0.87 (0.56 – 1.34)	---- ------------	--- -----------
				
*Health-related*				
-Activity of daily living^2^	Reference	1.17 (1.11 – 1.23)	Reference	1.20 (1.14 – 1.27)
-Impaired vision^2^	Reference	1.08 (0.94 – 1.23)	Reference	1.07 (0.93 – 1.22)
-Impaired hearing^2^	---- ------------	--- -----------	---- ------------	--- -----------
-Perceived general health (poor/fair)	Reference	1.07 (0.67 – 1.71)	Reference	1.32 (0.82 – 2.14)
-Number of chronic conditions	Reference	0.99 (0.87 – 1.13)	Reference	1.04 (0.91 – 1.19)
-Cognitive functioning^3^	---- ------------	--- -----------	---- ------------	--- -----------
-One or more falls in previous six months	Reference	1.49 (1.01 – 2.20)	Reference	1.31 (0.89 – 1.95)
				
*Psychosocial*				
-General self-efficacy^3^	Reference	0.99 (0.97 – 1.01)	Reference	1.00 (0.98 – 1.02)
-Mastery^3^	Reference	1.01 (0.96 – 1.06)	Reference	0.97 (0.92 – 1.02)
-Social support interactions^3^	---- ------------	--- -----------	---- ------------	--- -----------
-Feelings of loneliness (all the time/mostly/often)	Reference	1.26 (0.69 – 2.31)	Reference	0.73 (0.41 – 1.30)
-Feelings of anxiety^2^	Reference	1.03 (0.98 – 1.10)	Reference	1.05 (0.99 – 1.11)
-Symptoms of depression^2^	Reference	1.05 (0.98 – 1.13)	Reference	0.96 (0.90 – 1.03)

Note. Only significant demographic, health-related and psychosocial variables from Table 2 were included. Variables were entered simultaneously.

^1 ^Low education refers to elementary school, junior high school or lower vocational level. ^2 ^Higher level refers to poorer functioning. ^3 ^Higher level refers to better functioning

## Discussion

Our objective was to study the impact of an extensive range of socio-demographic, health-related and psychosocial variables on levels of fear of falling and avoidance of activity in older persons who avoid activity due to fear of falling. The results clearly show that due to the interrelationship between socio-demographic, health-related and psychosocial variables in older people, only a few of them correlated independently with either severe fear of falling and avoidance of activity due to this fear. Although we identified four socio-demographic, five health-related and five psychosocial factors significant univariate correlates of fear of falling, the independent correlation of only female sex, limitations in activity of daily living and falls history remained when covariates were taken into account. With respect to avoidance of activity due to fear of falling, we identified two socio-demographic, five health-related and five psychosocial significant univariate correlates; this pattern is consistent with fear of falling except for low education and living alone status being non-significant. When all significant univariate correlates were taken into account, only higher age and limitations in activity of daily living correlated independently with severe avoidance of activity. The correspondence between correlates for fear of falling and avoidance of activity may partly be explained by the interrelation of both outcomes. In effect, we were trying to determine what influences avoidance of activity in addition to fear of falling.

In a recent review Scheffer and colleagues [8] identified falls history, female sex and higher age as the main risk factors of fear of falling. In addition, decline in physical and mental functioning and quality of life as well as risk of falling were identified as the main consequences of fear of falling. These conclusions are partly similar to our findings in Table 2 when we compared older persons with and without *severe *fear of falling. However, in our final multivariate model where we took all significant univariate associations into account, only the impact of age, limitations in activity of daily living and falls history remained significant in the model. This latter finding indicates that *none *of the psychosocial factors we studied can differentiate between severe and mild levels of fear of falling.

Our study has several limitations. First, all measures were self-reported and performance-based measures were not included in the current study. Future studies should include objective measures of balance, gait, muscle strength, vision, etc. to establish a further understanding of the complex construct of fear of falling. Second, our study was cross-sectional so we may not take causal inferences from our data (e.g., between avoidance of activity and limitations in activity of daily living). Future research may identify which correlates can be considered as predictors and which as consequences of severe fear of falling and related avoidance of activity. Third, we included community-living older persons which can be considered as a heterogeneous sample. Future studies could focus on whether different patterns of correlates exist between more frail and less frail older persons. Finally, we included only persons who reported at least mild fear of falling and avoidance of activity. Also having people without fear of falling in our study would have enabled us to say whether, for example, women are more likely to develop any level of fear of falling or only severe fear of falling. However, our interest was not in the development of fear of falling, but rather in those factors that differentiate between mild and severe levels of fear and related avoidance behavior. From *clinical point of view *particularly older people with sever fear of falling and avoidance of activity due to fear of falling are relevant for interventions. Therefore it is important to identify the factors associated with severe levels of fear of falling and related avoidance behavior. Such knowledge may help to specify the contents, approach and length of interventions for these persons. So from clinical point of view it is relevant and important to distinguish between mild and severe levels of fear of falling.

The strength of our study is that in contrast to previous studies we focused on *severe *levels of fear of falling and avoidance of activity. We identified old age, female sex, limitations in activity of daily living, impaired vision, poor perceived health, chronic morbidity, falls, low general self-efficacy, low mastery, loneliness, feelings of anxiety and symptoms of depression as significant correlates of severe fear of falling and avoidance of activity. Although the knowledge about the unique associations of specific variables with levels of fear of falling is of interest for theoretical and scientific reasons, the awareness of the above univariate associations may help to specify the concepts for developing interventions and programmes to reduce severe levels fear of falling in old age, particularly in their early stages of development. A further strength of our study was the attempt to separate fear of falling and the resultant avoidance of activity as outcomes; particularly the latter is under researched in previous studies. This is important, as while fear of falling in itself may be an unwelcome anxiety for an older person, it is the related avoidance of activity that is arguably more significant in terms of its effect on social engagement, functional decline, quality of life, and further falls, particularly when this avoidance is severe. Our work suggests that avoidance of activity in older people with severe levels of fear of falling may be particularly high in those of advanced age and with limitations in activities of daily living, people for whom continued independence may be especially fragile and who will need rapid intervention to maintain that independence.

## Conclusion

We conclude that female sex, limitations in activity of daily living and one or more falls in the previous six months correlate independently with severe fear of falling. Higher age and limitations in activity of daily living correlate independently with severe avoidance of activity. Psychosocial variables did not contribute independently to the difference between mild and severe fear of falling and to the difference between mild and severe avoidance of activity due to fear of falling. However, old age, female sex, limitations in activity of daily living, impaired vision, poor perceived health, chronic morbidity, falls, low general self-efficacy, low mastery, loneliness, feelings of anxiety and symptoms of depression were identified as univariate correlates of severe fear of falling and avoidance of activity. Although the knowledge about the unique associations of specific variables with levels of severe fear of falling and avoidance of activity is of interest for theoretical reasons, the knowledge of univariate association may also help to identify older people at risk for severe levels of fear of falling and avoidance of activity (*secondary prevention*) but also to specify the concepts for developing interventions and programmes to reduce fear of falling and avoidance of activity in old age, particularly in their early stages of development (*tertiary prevention*).

## Competing interests

The authors declare that they have no competing interests.

## Authors' contributions

GIJMK designed the study as a part of a randomized controlled trial. GARZ and JCMvH collected the data. GIJMK analyzed the data and drafted the manuscript with contributions of KJM, KD, JCMvH and GARZ. All authors read and approved the final manuscript.

## Pre-publication history

The pre-publication history for this paper can be accessed here:


